# Organisational and Structural Drivers of Childhood Immunisation in the European Region: A Systematic Review

**DOI:** 10.3390/vaccines10091390

**Published:** 2022-08-25

**Authors:** Ronan Lemwel Valdecantos, Raffaele Palladino, Andrea Lo Vecchio, Emma Montella, Maria Triassi, Antonio Nardone

**Affiliations:** 1 Department of Public Health, University “Federico II” of Naples, 80138 Napoli, Italy; 2 Global Health Workforce Network (GHWN) Youth Hub, World Health Organization, 1211 Geneva, Switzerland; 3 Interdepartmental Center for Research in Healthcare Management and Innovation in Healthcare (CIRMIS), University “Federico II” of Naples, 80138 Napoli, Italy; 4 Department of Primary Care and Public Health, Imperial College, London SW7 2BX, UK; 5 Department of Translational Medical Sciences, Section of Pediatrics, University “Federico II” of Naples, 80138 Napoli, Italy

**Keywords:** vaccination coverage, national immunisation programme, strategic advisory group of experts on immunisation, mandatory immunisation policy, primary health care, expanded program on immunisation

## Abstract

Despite the implementation of widespread vaccination programs, the European Health Systems continue to experience care challenges attributable to organizational and structural issues. This study aimed to review the available data on aspects within the organizational and structural domains that might impact vaccination coverage. We searched a comprehensive range of databases from 1 January 2007 to 6 July 2021 for studies that reported quantitative or qualitative research on interventions to raise childhood vaccine coverage. Outcome assessments comprised organizational and structural factors that contribute to vaccine concern among pediatric parents, as well as data reported influencing the willingness to vaccinate. To analyze the risk of bias, the Ottawa, JBI’s (Joanna Briggs Institute) critical appraisal tool, and Amstar quality assessment were used accordingly. The inclusion criteria were met by 205 studies across 21 articles. The majority of the studies were conducted in the United Kingdom (6), the European Union (3), and Italy (3). A range of interventions studied in primary healthcare settings has been revealed to improve vaccination coverage rates including parental engagement and personalization, mandatory vaccination policies, program redesign, supply chain design, administering multiple/combination vaccines, improved vaccination timing and intervals, parental education and reminders, surveillance tools and Supplemental Immunisation Activity (SIA), and information model.

## 1. Introduction

The development and mass distribution of childhood vaccines has been one of the greatest public health achievements in history, underpinning marked progress in child survival and health outcomes worldwide [1]. Over the past four decades, global coverage of both longstanding and more newly available vaccines improved, and the number of zero-dose children have declined by nearly 75% since 1980 [2]. The 2011–2020 Global Vaccine Action Plan (GVAP) set forth various targets for childhood vaccination, such as reaching 90% coverage across all vaccines in National Immunisation Programmes (NIP) by 2020 [3]. However, 22.7 million children worldwide (17% of the target population) were not vaccinated with Diptheria-Tetanus-Pertussis3 (DTP3) in 2020, although this figure improved as compared to the previous year (14%) [4]. In 2016, vaccination coverage was below 95% (i.e., herd immunity-target threshold at least for most vaccines) in 22 out of 29 European Union (EU)/European Economic Area (EEA) countries for the second dose of a measles-containing vaccine (MCV) according to the most recent data collected [5]. In Southern Italy, for example, in the same year, only 77.8% of children were appropriately vaccinated for their age with a measles-containing vaccine [6]. The COVID-19 pandemic has dramatically impacted paediatric vaccination coverage [7,8]. Full recovery from COVID-19–associated disruptions will require targeted, context-specific strategies to identify and catch up on zero-dose and under-vaccinated children, introduce interventions to minimize missed vaccinations, monitor coverage, and respond to program setbacks [9].

Differences in vaccination coverage within EU member states can be partially explained by the regional differences. Each EU Member state has its own history, characteristics, and habits. The National Health Services (NHS) of most of these countries have diverse vaccination systems, different vaccine recommendations, and unique schedules of vaccine administration—which means that immunisation is not considered in the same way and, at least for some antigens, vaccination coverage does not always meet changing medical needs [9]. Indeed, no two NIP are alike [10,11]—within the EU/EEA, countries vary considerably with respect to recommended vaccines, organization of health services, the mandate of public health agencies, legislation on confidentiality, and other relevant factors [12]; and not all European countries are assured assistance under these tried-and-true intervention plans. However, according to a study, with the increasing globalization of vaccine development, licensing, and marketing, it might appear a logical consequence that immunisation schedules should therefore become more uniform [13]. Recent data from the European Centre of Disease Prevention and Control (ECDC) show that 12 member states feature at least one vaccination that is mandatory; and with the exception of Belgium, these countries provide a range of vaccines that are part of a mandatory schedule [10]. Across the region, state-sponsored mass vaccination programs have repeatedly led to heated political debate, whether about pertussis vaccination (against whooping cough) in the 1970s [14], the measles, mumps, rubella (MMR) vaccine, the vaccine against H1N1, Hepatitis B [15], or the vaccine against Human Papilloma Virus (HPV) [15].

Evidently, increasing vaccination program resources is always a challenge for a number of national healthcare systems [16]. Meanwhile, a study has highlighted a number of interventions that can help improve childhood immunisation rates in developed countries [17]. These include reminding parents and providers of upcoming and overdue immunisations, and educating and providing feedback to the vaccination providers [18]. Although the physician-targeted communication intervention had no effect on maternal vaccine hesitancy or physician self-efficacy, there is a need for further research to recognize physician communication strategies that are effective in reducing parental vaccine indecision in the primary care setting [19]. Further investigation is also warranted into the perceptions of providers and parents, particularly regarding the use of social networking sites and strategies on how to overcome providers’ reluctance to adopt newer technologies for the purpose of immunisation reminders [20]. Additionally, there is insufficient evidence to guide effective strategies for dealing with the emerging threat of parental vaccine refusal [21]. As parental vaccine hesitancy co-exists within the vaccine records; gaps on immunisation coverage and estimates cannot be regarded as a reliable indicator of vaccine hesitancy [22]. Hence, the priority is to address the factors that limit the more important accessibility and availability of vaccines [23], and it is not the only key to success. The structure of healthcare systems as well as the contacts and relationships they establish with their populations appear to be determinants. The local vaccination culture that emerges because of this interaction may hold the key to explaining the differences observed between health regions and systems [23]. To address this growing phenomenon, it is critical that we understand what approaches are effective, and there should be a greater emphasis on preventing vaccine uncertainty to reach the favourable vaccine immunisation targets [24]. Interestingly, it has been determined by the World Health Organization (WHO) Strategic Advisory Group of Experts on Immunisation (SAGE) that low vaccine coverage prevails due to flaws in vaccine availability such as stock-outs, infeasible travel/distances to reach immunisation clinics, missing vaccine program communication, or curtailment of vaccine services due to conflict, a natural disaster, or other disruption [25].

According to the SAGE collaboration, “recent outbreaks should be warning signs against complacency”. For instance, the United Kingdom (UK) has seen a successful campaign to add pneumococcal vaccination to the primary schedule with a catch-up campaign for older children. This has resulted in a substantial reversal of previously increasing trends with falls in hospital admissions for bacterial pneumonia (20%) and empyema (22%) in 2 years after implementation, linked with uptake rates of 80% and 98% after the first and second years of the campaign, respectively [26]. To avert the worst occurrence, using appropriate surveillance tools, computerized immunisation registries collect data on individual vaccines into a nationwide database, allowing coverage monitoring and planning to maintain and enhance coverage [27].

Given the growing global concern and long lines of challenges over childhood vaccine strategies and hesitation, the current study focuses on the organizational, structural areas and encounters that must be addressed in order to maintain targeted vaccination coverage in the WHO European Region [28]. Specifically, this systematic review study aims to: (i) identify and synthesize the Organizational and Structural Institutionalized procedures, policies, and health care practices that impede or influence the success of European Nation Immunisation Programs for children aged 0–6 years. (ii) Thoroughly assess and justify the reasons for vaccine apprehension among parents and guardians of pediatric patients in private and public healthcare institutions.

## 2. Methodology

### 2.1. Criteria for Inclusion and Exclusion

There were three basic requirements for inclusion: (1) studies published in English that reported original research, such as cohort studies, cross-sectional studies, qualitative investigations, literature reviews, ecological studies, and systematic reviews and meta-analyses; (2) children under the age of six who lived in the WHO European Area were our target group; (3) studies that reported on our primary outcome measure: the proportion of the target population who had received all of the recommended universal childhood immunisations. The results could be for single immunisations or a combination of vaccines that are due. We did not include research where the whole paper was not available or studies that did not contain any original data, such as conferences, editorials, or letters.

### 2.2. Search Strategy

We systematically searched electronic databases (Cochrane CENTRAL, MedLine, EMBASE, PsycInfo, Psychology and Behavioral Sciences and CINAHL) for articles published from 1 January 2007 to 6 July 2021. We included only relevant studies published in the last 15 years. Keywords and Boolean operators were used for search strategy and tailored to each database, as reported in detail (see Table 1). keywords for identifying preventive health care using MeSH and key terms including “primary care”, “primary health care”, or “primary healthcare”; keyword for identifying organisational domain “organisational structure”; keywords for identifying pre-school vaccination were “child*”, “childhood immunity*”, ‘vaccine*”; keywords for identifying the setting were “developed countries”, “developing countries”, “European Union”, “EU”, and “Europe” to identify studies reporting interventions to improve childhood immunisation coverage and to evaluate their effectiveness in children in the European region. To locate more papers, we hand-searched the reference lists from the collected studies and reviews and contacted experts in the field. However, we excluded grey literature including conference papers, dissertations, and government directives, concentrating instead on authenticated peer-reviewed literature.

**Figure 1:**
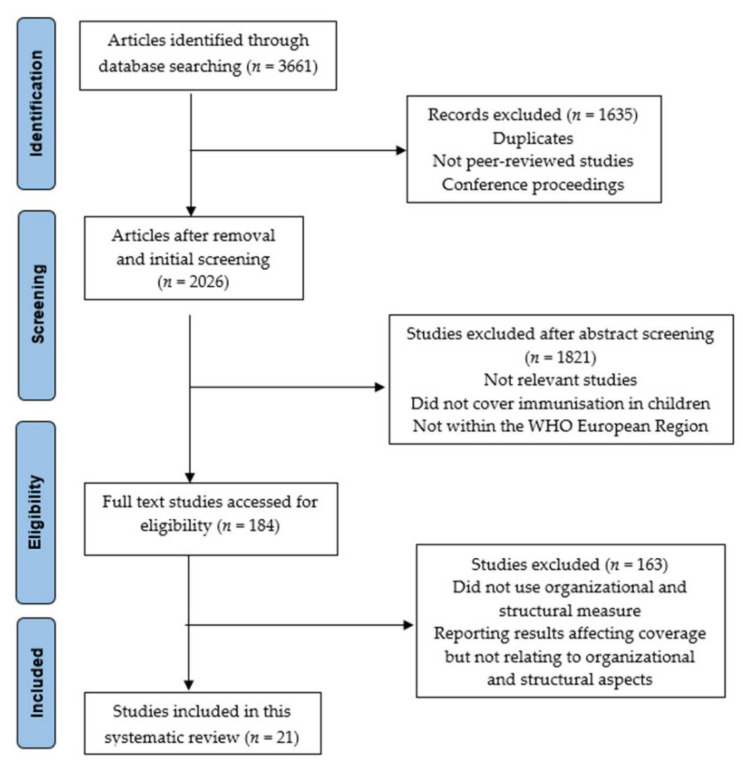
PRISMA flow diagram illustrating the search strategy and study process of selecting.

The PRISMA diagram (Figure 1) shows the selection process and the grounds for exclusion. Initially, the titles and abstracts of all publications found through a search were screened by a single investigator. All studies that did not pertain to organizational and structural domains impacting children’s immunization coverage were omitted based on their titles and abstracts. Full-text articles that qualified were acquired. The relevance of these full-text articles to the relationship between organizational and structural constructs and vaccine coverage was verified after appraisal.

This systematic review was carried out in accordance with the Preferred Reporting Items for Systematic Reviews and Meta-Analyses (PRISMA) guidelines [29]. The electronic searches yielded a total of 3661 studies, of which 1635 and 1821 studies were eliminated after analyzing their titles and abstracts, respectively. For a full-text review, the remaining 205 publications were retrieved. There were a total of 21 papers that matched the requirements for inclusion (Figure 1). Geographic location, age group, or outcomes of interest, as well as inadequate study design, were the most common grounds for exclusion.

### 2.3. Data Extraction and Quality Assessment

A single reviewer independently checked all citation titles and abstracts for eligibility, using Rayyan Artificial Intelligence [30] to find those that met the inclusion requirements. All duplicates were removed, and if the abstract did not provide enough information to determine eligibility, the entire paper was retrieved for further assessment. Skepticism about the articles was resolved by discussion with the involvement of another reviewer.

Additionally, a lone reviewer independently extracted data from included studies on study setting, participant characteristics, healthcare setting, interventions, and outcomes measured. Included studies were scored for methodological quality using Ottawa [31], JBI’s critical appraisal tool [32], and Amstar [33], appropriately depending on the study design.

## 3. Results

### 3.1. Characteristics of Included Articles

Characteristics of the 21 selected studies are summarised in Table 2. The 21 articles selected were published between 2012 and 2021 [12,34,35,36,37,38,39,40,41,42,43,44,45,46,47,48,49,50,51,52,53]. Three of the 21 studies were published between 2012 and 2014 [46,49,52], nine between 2015 and 2018 [35,37,38,41,42,43,47,48,53], and nine between 2019 and 2021 [12,34,36,39,40,44,45,50,51]. Ten cohort studies [38,39,41,42,43,45,46,47,51,53], six cross-sectional studies [37,40,44,48,49,52], two qualitative studies [34,35], one ecological [50], one narrative literature review [36], and one systematic and meta-analysis study [12] were included. The majority of the studies were carried out in the United Kingdom (6) [35,41,42,44,51,53], the European Union (3) [37,42,50], and Italy (3) [39,48,52], with the remainder conducted as combined-diverse studies in England, Israel, and Sweden (1) [34], Armenia and Kyrgyztan (1) [47], and as country studies in France (2) [38,40], Denmark (1) [45], Greece (1) [49], Norway (1) [36], Switzerland (1) [46], and the Czech Republic (1) [43] (Table 2). The study participants were from 0 to 6 years of age, with the majority of studies focusing on preschoolers. Studies were conducted in paediatric outpatient clinics, family practices, primary care clinics, community health centers, managed care organizations, health maintenance organizations, and community clinics throughout the WHO European Region.

Table 3 summarises the study characteristics within a general practice setting. The interventions in our review were parental engagement and personalization, mandatory immunisation policy, remodeling the vaccination program, procuring and distributing vaccines, administering multiple/combination vaccines, improving immunisation timing and intervals, parental education and reminders, surveillance tools and supplementary immunisation activity, and information technology (Table 3).

### 3.2. Parents’ Engagement and Personalisation Intervention

Only one paper on parents’ engagement (celebration card-based intervention) and one study on personalization involvement (population-level interventions) that examined parental reminders and motivation were included. Using the JBI Qualitative Study Quality Scoring, the first study received a quality score of 9 out of a possible 10. Celebration card is a program aimed at informing parents about impending vaccines and encouraging parents of children who are late. Furthermore, the Celebrate and Protect program is a collaborative effort that engages parents and caregivers to build relationships with service providers and, as a result, increase immunisation uptake. Researchers should continue to experiment with strategies that focus on engaging parents with thoughtfully presented, evidence-based information, as parents’ engagement and intervention strongly suggest personalisation. The second study found that when standardized population-level interventions were compared to individualized health or welfare interventions, frontline workers should be equipped with the skills and resources necessary to increase parents’ compliance during direct-delivery contacts that scored a perfect ten on the JBI scale. Successful interventions will be those that foster trust, alleviate parents’ concerns about unfounded vaccine risks, and assist parents in understanding that vaccinating on schedule is in the best interests of everyone, including their own children [55].

### 3.3. Mandatory Immunisation Policy

In the systematic review, using appropriate quality assessment tools, we looked at five studies about Mandatory Immunisation Policy (MIP)—three of them were high-quality studies, one was middle-quality, and one was low-quality. Higher vaccination coverage was linked to mandatory vaccination and the severity of the penalty. Furthermore, mandatory vaccination without nonmedical exemptions was linked to a reduced incidence of vaccine-preventable diseases in countries with mandatory immunisation. According to a study among high-income countries, current vaccination policies are not sufficient to achieve and maintain measles elimination in most countries. Strategies targeting unvaccinated children before they enter primary school can remarkably enhance the fulfillment of WHO targets [56]. In July 2017, France announced significant revisions to its vaccination policies, with children being required to be vaccinated beginning in 2018 [38]. Only three immunisations are mandated at this time: diphtheria, tetanus, and polio. All 11 childhood immunisations presently recommended by health authorities will be made mandatory under new legislation [57]. This legislation follows Italy’s announcement in May 2017 that all children aged 4–16 years would be obliged to get 12 recommended immunisations, including for the hepatitis B virus, in order to attend school, with substantial fines for non-compliance [58].

### 3.4. Vaccination Program Remodeling

The two Vaccination Program Remodeling studies included in the systematic review attained an Ottawa Quality Assessment for Study score of 8 out of 10, resulting in an expansion of the present vaccination schedule to include Hepatitis B and/or Men B, maximizing a general practitioner (GP) appointment with the amount of desired injectable vaccinations; reducing a future immunisation-dose-visit and limiting any foreseeable missed vaccinations [41]. Another included UK-related study, demonstrating the feasibility and utility of the Vaccination Remodeling approach, found that the four investigated schedules differed by at most 19% in terms of residual disease burden expressed in quality-adjusted life year (QALY) loss; it was realized that the largest residual of disease is related with measles and pneumococcal infection, according to each schedule [44].

### 3.5. Vaccination Procurement and Distribution

The three included studies on procurement and distribution received an 8 out of 10, with one included study of increased use and procurement of serotype vaccine demonstrating a significant reduction in Invasive Pneumococcal Disease (IPD) in the Czech Republic [43], through the Ottawa Quality Appraisal scale for a cohort study. In addition, having the same quality scoring, an included study in England and Wales found a lower immunisation probability among larger families with eligible children [42]. Additionally, according to a study conducted in the United Kingdom, if immunisation can begin without significant delays, strategies based on responsive vaccine purchase offer a greater advantage than plans based on the procurement and maintenance of a stockpile [44]. IPD incidence in children is being reduced further through universal administration and distribution using broader serotype coverage rather than non-vaccine serotypes, as well as increased vaccination rates [59].

### 3.6. Combination/Multiple Vaccine Administration

One study included in the review discussing administering multiple/combination vaccines received an 8 out of 10 using Ottawa quality scaling. The recommended minimum interval between DTaP-IPV-Hib-2 and DTaP-IPV-Hib-3 was generally followed by vaccination providers [45]. However, another finding indicates that the reluctance to administer more than three injections in a single GP visit during infancy could be a problem [53]. Evidently, providing DTaP-IPV-Hib-3 and MMR-1 at the same time resulted in low Danish Nationwide Register compliance [45].

### 3.7. Improved Immunisation Timing and Intervals

Two included studies scored high on immunisation timing and intervals from the Vaccination Programs in the systematic review. One study quality score was 9 out of a possible 10 using the Ottawa Scale for Cohort Study. This outcome implies that the said effectiveness evolved in both countries (Armenia and Kyrgyzstan), resulting in a significant increase in immunisation coverage over the last few decades [47]. In reality, the proportion of children in Armenia with a correctly timed three-dose vaccination of DPT increased from 75% (1997) to 87% (2012) and the proportion of children in Armenia with a correctly timed first dose of DPT-increased from 46% (2000) to 66% (2010) [47]. On the contrary, in a group of children aged 6 months to 2 years who were reflecting the age distribution in the cohort study, effective vaccine coverage of measles was lower [46].

### 3.8. Parental Education and Reminder

Two of the evaluations looked at the efficacy of basic parental education programs in terms of vaccination. One study examined immunisation coverage and discovered that a child’s age was a significant predictor of vaccination completion, while another examined new immunisation information and verbal explanations and discovered that they received a 9 out of 10 quality rating and an 8 out of 10 quality rating, respectively. Both of these studies exhibited a significant influence on vaccination rates and coverage, underlining the need for creating an efficient vaccine reminder system, particularly for newly released vaccinations [48,49]. This review looked at educational programs that may be given legally in the setting of primary care. However, giving information or education to allow parents or guardians, as well as communities, to make educated health decisions is a crucial component of all health systems [60].

### 3.9. Surveillance Tools and Supplementary Immunisation Activity (SIA)

Two included studies were about surveillance tools and SIA, one of which was conducted in the UK and Northern Ireland and received an 8 out of 10 on the Ottawa Appraisal tool for Cohort study. Our assessment found that two primary care data sets about surveillance tools were insufficient for reliably assessing vaccination coverage and revealed that the triangulation routine data revealed little additional immunisation activity. For instance, when a 25% coverage underestimation adjustment factor was used in the sensitivity analysis rather than 50%, susceptibility estimations indicated that no birth cohort between 1989–1990 and 2006–2007 attained a sufficiently high degree of immunity to prevent measles transmission [51]. Moreover, using a public health monitoring instrument, an included ecological study scored 10 out of 16 on the AMSTAR Quality Appraisal—averages for vaccine uptake indicated substantial heterogeneity between European area nations. For example, DPT3 uptake varies significantly by nation, ranging from 86.26 percent in Romania to 99.87 percent in Hungary [50].

### 3.10. Information Technology

The two studies looking at the progress in the child health information model received high marks in our quality evaluation. Limitations in technological availability and advances in service delivery and record-keeping were recognized and agreed upon in these investigations. In fact, only six of Italy’s 15 completely computerized regions can determine vaccination coverage automatically [52]. Additionally, new research is increasingly demonstrating the potential benefits of novel approaches such as text messages to transform how reminder/recall is implemented [61].

## 4. Discussion and Implications

Policymakers and healthcare managers in the European Region must use sound judgment in determining the most efficient use of resources available to preserve and enhance healthcare quality in order to maintain and/or increase targeted vaccination coverage. When making such decisions, they must look at the potential areas for improvement initiatives in primary health care (PHC) activities from both structural and organizational perspectives, the plausible economic impacts of introducing new quality improvement interventions, and the expected potential benefits of any changes in healthcare worker behavior. Our systematic review study found that including parental engagement and personalization, mandatory vaccination policy, vaccine program redesign, vaccine procurement and distribution, administering multiple/combination vaccines, improved vaccination timing and intervals, parental education and reminders, and administering concurrent vaccinations could be effective in improving Expanded Program on Immunisation (EPI) performance specifically in the WHO European Region. When determining which combination of interventions to use for which community or at what time period, policymakers and decision-makers would need to determine intervention strategies that might help raise childhood vaccination rates.

Parents’ engagement and personalisation interventions to promote primary vaccination uptake in children have been described in a number of studies. Parental participation through the use of celebration cards is an effective strategy. Participants’ perceptions of celebration cards were more of a reminder than a “call for action”, which may be perceived as a secondary reminder, according to responses from the focus group and some providers. In one intervention study, according to a comparative examination of street-level negotiation, nurses in all three nations (England, Israel, and Sweden) responded to compliance by participating in negotiations with parents, which is affected by institutional alternatives accessible to health care providers in their compliance efforts [34]. In Greece, total immunisation rates for new vaccinations (Men C, PCV7, varicella, Hepatitis A) were lower, ranging from 61% to 92%, indicating that parental reminders are needed [49]. This outcome could be due to a lack of awareness, new vaccine efforts, or a combination of factors. Interestingly, a relevant study reiterated that involving communities and relevant leaders in immunisation programs can be very effective [34] However, the lines of responsibility and the authority to determine and execute different measures need to be clarified to ensure that such measures are linked with national health policy and do not deter underserved families from vaccinating their children [62].

MIP comparative analysis on two periods (2006–2010 and 2015–2017) has shown that, for non-vaccination in children, there was an increase from a mean of 3.4% to 5.5%, after the national immunisation policy arms had been lifted in the later period [37,63]. In a study collecting data from 31 European countries, the totality of participants was in favour of vaccinations in childhood, eleven nations of which introduced mandatory vaccination (35.4%), while the others strongly recommended vaccination [37]. This likely represents a strong intention among European national leaders to promote the healthy and free mobility of their citizens in the direction of enormous globalization throughout the European region. In fact, following the infant mandatory immunisation in 2018, the proportion of mothers in favour of vaccination increased significantly—Hepatitis B (Hep B) and Meningococcal C (Men C) vaccination coverage rates significantly progressed between 2017 and 2018 [38]. Similarly, vaccination coverage for the first dose of MenC has increased by 36.4 percent among infants in France during the same period, resulting in an almost threefold drop in MenC cases compared to the preceding five years. On the contrary, notable recommendations from a study include unanimous support for mandatory childhood vaccination in Ontario, the need for broad educational communication about mandatory and optional vaccination (vaccines that are not included in the immunisation program due to concerns about side effects, high costs, and a lack of information) [64], and the development of a no-fault compensation scheme for Adverse Events Following Immunisations (AEFIs) [65]. This upheaval around multi-stakeholders may arise from debatable issues and pointing fingers about taking AEFIs responsibility. Remarkably, between the 2000 and 2012 surveys in Armenia, the most significant rise in correctly timed vaccines was recorded for MCV, which increased by 59 percent from 39 percent to 62 percent. In Kyrgystan, after three years of evaluation, a similar pattern was noticed, with a 12 percent increase [47]. However, due to policy changes in Switzerland, 66.5 percent of children aged 9 to 12 months were found to be susceptible, despite a cohort study showing a 20 percent early uptake of MCV1 between 9 and 12 months [46]. These data can be used to help shape legislation aimed at raising immunisation rates.

Using Information Technology (IT), the European Commission, member states, and child health stakeholders agreed to make progress in developing child health information models and digital health standards, as well as identifying areas that require further standardization and desirable steps toward innovation in service delivery and record-keeping [66]. Only eight of Italy’s 21 regions receive data in real-time; three receive it quarterly, and five receive it annually [52]. The ability of various systems to manage vaccination coverage data at the regional level varies greatly; only six of the 15 completely computerized regions can calculate vaccine coverage automatically [67]. Only three of these six can calculate coverage using individual data from the Local Health Units (LHUs) in real-time. Relative to an increasing information technology need, research implied that commercial viability will depend on healthcare policy/public acceptability of microneedle technology [52]. An effort must be made to identify the barriers to acceptance and overcome them by increasing awareness and education in stakeholder groups pertaining to the paediatric population [68].

Increasing education among more than half of the parental group lead to an expressed desire to learn more about immunisations for their children [48]. Parents who were unsure if they were following the recommended immunisation schedule and if it was a good idea for their children, as well as parents of first-born children, were more likely to require additional information. This discovery may necessitate increased maternal education and reminders, particularly among prime mothers, as well as tailored intervention. Findings from a systematic review and meta-analysis suggested that several robust interventions, particularly postal reminders, combined recall and reminder strategies, and discussion-based education, can increase childhood immunisation coverage [69]. Indeed, research highlights the challenges parents encounter when choosing whether or not to vaccinate their children [48].

In administering multiple/combination vaccines, contributing variables according to the Danish study included in this systematic review could be vaccination providers’ aversion to giving numerous injections and a preference for sticking to the immunisation sequence in the vaccination program; more work is needed to increase vaccine timeliness and coverage [45]. Backing up the results of the present study showed that Adverse Event Following Immunisation (AEFI) was very rare; the vast majority of them were non-serious and, despite the claims of anti-vaccination movements, the simultaneous administration of vaccines was safe and did not influence the risk of reporting a serious AEFI, particularly in children [70]. Additionally, according to the study findings, fully using all possibilities for simultaneous delivery of all age-eligible vaccine doses during the same immunisation visit is crucial for meeting the 95 percent MCV2 coverage objective. Future treatments concentrating on the group with risk factors identified could significantly reduce missed opportunities (MO) for simultaneous MCV2 injection, to enhance MCV vaccination coverage, and ultimately meet the objective of eliminating measles [71]. Interventions with parents of partly vaccinated children should prioritize reducing obstacles to reaching community health professionals in order to achieve a schedule completion on a directed basis [72]. Indeed, vaccination data on long-term immune response durability, immunological memory, and vaccine efficiency are highly anticipated in a timely manner, as is recombination [73].

Regarding vaccine procurement and delivery, if immunisation can begin without significant delays, plans based on a responsive vaccine purchase have a greater benefit than plans based on the purchase and maintenance of a stockpile [44]. Ironically, this is not predicated on a responsively bought vaccination being more effective than a stockpiled vaccine, but rather on avoiding the expense of maintaining and refilling a reserve. Meanwhile, according to a study conducted in England and Wales, children living in areas of higher disadvantage and in larger families are less likely to obtain the influenza vaccine [42]. Finding out whether techniques like providing vaccines in diverse locations may increase vaccination uptake among children, particularly in underprivileged populations, is critical. Furthermore, research done in the Czech Republic found that a higher vaccination rate and a single, more comprehensive vaccine for regular childhood immunisation may reduce IPD in children [43]. While the structure and operations of immunisation programs differ between countries, relevant authorities should do more research to identify areas that may need redesigning to make the vaccine supply chain system more efficient and effective to achieve the strategic priority goals of the immunisation agenda 2030 [74]. For instance, to enhance vaccination administration, appropriate regulations, vaccine sourcing, purchasing, logistics, and techniques should be adopted for distinct areas and regions [75].

A UK study illustrates the potential value of analyzing the program-wide effect of vaccination schedule changes, and a newer framework is a vital step toward building a technique for doing so consistently [53]. In another piece of UK research, vaccination program remodeling is potentially beneficial in evaluating the program-wide impact of adjustments to an immunisation schedule has been established and our framework is a significant step toward developing a method for doing so, systematically [41]. A structured method was proposed to derive intervention scenarios from the conceptual Immunisation System (IMS) diagram. Probably, a newly designed framework can be applied as a problem structuring tool, and as part of the system design process [76].

Using a surveillance tool and supplemental vaccination activity in areas with a higher prevalence of vaccine reluctance, a study conducted in the European Union found statistically significantly lower regional vaccination immunisation rates [50]. Eventually, for instance, researchers were able to get new insights into population immunity and identify susceptible populations by merging routine data on measles vaccination coverage with disease monitoring, which aided in prioritizing public health actions aimed at closing immunity gaps. A collaborative study also emphasized the need to integrate the requirement for district-level coverage estimates with the operational and cost consequences of surveying district-level representatives [77]. According to the WHO, it is important to develop a monitoring and evaluation (M and E) plan at the same time as the intervention is being designed, and implementation is being planned [78]. Survey tools are critical for evaluating SIA coverage, which is especially prone to inaccuracy due to the lack of time available to verify the age or residency of those arriving for vaccination or to appropriately record and report immunisations. For routine vaccination coverage, the multiple indicators cluster survey (MICS) and demographic and health survey (DHS) will usually be sufficient to monitor trends at national and often also provincial levels [79].

## 5. Strengths and Limitations

Our search strategies turned up more studies than any previous review of its kind. We combined studies from a variety of clinical settings and socioeconomic demographics in industrialized countries, allowing us to generalize the findings in this context. Many developed countries have ample practitioners who provide universal access to primary care services. To guarantee the broadest possible range of studies and to avoid publication bias, the scoping review that includes different databases published in peer-reviewed journals were searched. Hence, we excluded studies outside the WHO European Region because health systems organization might differ profoundly, translating into different barriers, including financial barriers and at a population-to-health workers level that is generally irrelevant to parents and general practitioners in many developed countries that provide universal access to primary care services. The settings, service delivery, intervention delivery, and quality of the included studies were all different, making meta-analysis challenging. Additionally, we did not include studies written in languages other than English, which is the study’s fundamental flaw. Finally, since a single individual was responsible for data search, data extraction, and risk of bias assessment, human mistake or experimenter bias cannot be ruled out.

## 6. Policy Implications and Future Research

The impact of general practitioner financial incentives and healthcare workers commensurate on vaccine uptake was not investigated. These disparities are assumed to be due to inclusion criteria leaning towards our systematic review study. Vaccination interventions have a stronger impact on individuals who are most at risk of being under-vaccinated. As a result, it is critical that vaccine coverage statistics be gathered in a way that shows disparities in uptake rates within socioeconomic groups, as well as between the general public and healthcare providers. In creating a bigger picture, these studies indicate the potential benefits of evaluating the program-wide impact of immunisation schedule modifications, and framework modifications can undoubtedly help improve vaccination programs and coverage over time. There is a need for further research to investigate alternatives to traditional policy reform. We may need to redesign and rebuild from the ground up [80].

## 7. Conclusions

Maintaining high vaccine uptake rates is critical to the success of any vaccination program and the improvement of children’s health. Parents and the general public must be actively engaged by health planners and specialists, and process mechanisms must be implemented to guarantee that children receive primary prevention. A number of measures have been identified in our systematic review study that can help boost childhood immunisation rates in the WHO European region. These include Parents’ Engagement and Personalisation Intervention, Mandatory Immunisation Policy, Vaccination Program Remodeling, Vaccination Procurement and Distribution, Administering Multiple/Combination Vaccines, Improved Immunisation Timing and Intervals, Parental Education and Reminder, Administering Multiple/Combination Vaccines, Vaccination Procurement and Distribution, Vaccination Program Remodeling, and Surveillance Tool and SIA. Hence, more research is needed to determine the effectiveness of these interventions and their effects on low vaccination rates for vaccine-preventable diseases in children.

## Figures and Tables

**Table 1:** Search-related keywords and Boolean operators.

Search	Search Terms
1	“vaccine hesitancy” AND (“European Union” or EU or Europe) AND (child or pediatric or paediatric or kids)
2	(“primary care” or “primary health care” or “primary healthcare”) AND (“childhood immunization” or vaccine) AND (“developed countries” and “developing countries”)
3	(“vaccine hesitancy” or “vaccine refusal”) AND (“European Union” or EU or Europe) OR organizational structure in healthcare AND (child or kids or pediatric)
4	“vaccination coverage” AND (“European Union” or EU or Europe) AND (child or pediatric or paediatric) AND (immune and vaccine)

Search terms: vaccination coverage, vaccine hesitancy, European Union, Europe, child, pediatric, kids, primary health care, childhood immunization and vaccination, developed and developing countries, organisational structure in healthcare.

**Table 2:** Characteristics of selected articles (*n* = 21).

No. of Papers	*n* = 6	*n* = 3	*n* = 3	*n* = 1	*n* = 1	*n* = 1	*n* = 2	*n* = 1	*n* = 1	*n* = 1	*n* = 1	*n* = 21
Characteristics	UK	EU	Italy	England, Israel, Sweden	Armenia and Kyrgyzstan	Denmark	France	Greece	Norway	Switzerland	Czech Republic	Total
Publication year												
2012–2014			1					1		1		3
2015–2018	3	1	2		1		1				1	9
2019–2021	3	2		1		1	1		1			9
Study Design												
Cohort	4		1		1	1	1			1	1	10
Cross-sectional	1	1	2				1	1				6
Qualitative	1			1								2
Narrative									1			1
Ecological		1										1
Systematic and Meta-analysis		1										1
Quality Assessment												
High	5	2	3	1	1	1	2	1		1	1	18
Intermediate	1	1										2
Satisfactory									1			1

For better quality scoring purposes, the included studies were scored using three appraisal tools for methodological quality using Ottawa [31] (for cohort and cross-sectional studies), JBI’s critical appraisal tool [32] (for narrative and systematic review and meta-analysis), and Amstar [33] (for ecological), appropriately employed depending on the study design.

**Table 3:** Study characteristics included articles influencing vaccine coverage.

*Study Characteristics of Parents’ Engagement and Personalisation Intervention*						
Study	**Quality**	**Type of Study**	**Setting/(Country)**	**Population**/ **(Subject)**	**Intervention/Method**	**Result/Outcomes**
Gofen, 2019	10/10	Qualitative Study	Backwords mapping Approach/England Israel and Sweden	Personalizing Immunisation	Two questions guided this study: how do frontline immunisation nurses encounter and respond to parents’ noncompliance with childhood immunisation; that is, how, and to what extent, do nurses exercise their street-level discretionary power to secure parental compliance with children’s immunisation? what role do regional and national public health officials play in the compliance efforts exercised by the nurses?	In all three countries, street-level negotiation emerged as a similar three-phase process: a trigger that starts the negotiation as parents introduce doubts whether or not to comply with the immunisation protocol. Notably, parental concerns are almost always portrayed by interviewees as legitimate; reciprocal discussion, which entails an exchange of perceptions, attitudes and professional information between parents and nurses; and a negotiated outcome, in which street- level discretion is exercised to adjust the rather strict delivery protocol to a more personalized immunisation provision.
Lwembe, 2021	9/10	Qualitative Study	Celebrate and Protect program/UK	Children under 5 years old	Engaging with parents and carers of children in order to improve the relationships between service users and providers thru data collection from conducting semi-structured telephone interviews or focused group.	Responses from the focus groups (and some providers) indicated that the participants’ perceptions of the celebration cards were more of a reminder than a ‘call to action’.
*Study Characteristics of Mandatory Immunisation Policy*						
**Study**	**Quality**	**Type of Study**	**Setting** **/(Country)**	**Population/** **(Subject)**	**Intervention/Method**	**Result/Outcomes**
Gamlund, 2020	Low quality	Narrative Literature Review	Norway	Arguments against introducing a mandatory childhood vaccination programme	Potential arguments that justify the benefits of mandatory immunization: autonomy harm principle herd immunity parental rights precautionary approach	Three arguments justified the potential benefits of mandatory immunisation that outweigh the disadvantages: harm argument herd immunity Precautionary Strategy
Bozzola, 2018	7/10	Cross-sectional study	EU	Mandatory vaccination policies in European Union	Policies of mandatory or recommended vaccinations of the European Countries gathered by ECDC compared to Italian guidelines.	(1) Countries introduced mandatory vaccination (35.4%) and the other recommended Eleven vaccination. Latvia has ten mandatory vaccines in childhood as well as Italy. (2) All the European Countries recommended or introduced compulsory vaccinations for the following vaccinations: tetanus, diphtheria, pertussis, Haemophilus influenza type B, Hepatitis B, poliovirus, mumps, measles, rubella with the exception of Iceland that did not recommend Hepatitis B vaccination.
Levy-Bruhl, 2019	8/10	Cohort study	National Social Security Reimbursement Data/France	Children under 2 years	Assessing the potential consequences of changes on mandated vaccination coverage extension of recommended vaccines thru data collection: (1) Child health certificates mandatorily filled at 24 months (2) Virtually, 100% of reimbursements of vaccines delivered in a given month are available two months later in the database	The increase in MMR first dose and MenC second dose vaccination coverage between 2017 and 2018 was 3.0% and 5.7%, respectively. This compared with a 0.3% and 3.6% increase between 2016 and 2017 respectively.
Gianfredi, 2019	8/10	Cohort Study	Regional data/Italy	Children 24 months old and younger	Electronically developing data in Microsoft Excel^®^ for VC collection that contains a specific section named “Reasons for no or incomplete vaccination against polio and measles” that includes a list of pre-defined reasons, updated in 2014, which consists of: emigrated to a different Local Health Unit or abroad excused permanently for health conditions or other causes not found because nomadic or homeless not found although known address temporary informed dissent definitive informed dissent immigrants who are waiting for their vaccination certificate from their own country or immigrants who re-start but did not complete the vaccination schedules; acquired immunity subsequent to previous disease or vaccination performed elsewhere found/contacted, but did not attend the appointment others reasons without further.	Percentage difference of missed vaccination with a specific reason from “other reasons without further details”; approx. 80% in 2006–2010 received Polio non-vaccination reason compared to more than 90% between 2015–2017 and historical non -vaccination mean of 3.4% and 5.5% in respective periods.
Martinot, 2021	9/10	Cross-sectional	Implementation of vaccine policy and its effectiveness/ France	0 to 35 months old	Internet-standardised questionnaire; mothers answered based on opinion on vaccination and vaccinations recorded in their child’s health record.	69% of mothers were in favour of vaccination while this rate dropped from 80.2% in 2012 to 64% in 2017, and 80.8 to 89.6% perceived Hep B, Men C measles and pertussis percentage vaccinations as useful/essential, in progress versus 2017.
*Study Characteristics of Vaccination Program Remodeling*						
**Study**	**Quality**	**Type of Study**	**Setting** **/(Country)**	**Population/(Subject)**	**Intervention/Method**	**Result/Outcomes**
Crowe, 2015	8/10	Cohort study	A Novel Framework/UK	2 to 60 months old aged children/developing a modelling framework and estimate the effective coverage against all Vaccination Preventable Diseases within an Immunisation schedule	Estimating the effective coverage against all diseases within a schedule through Modelling Approach.	(1) Introducing Meningitis B vaccination could saturate the early (2-month) visit, thereby potentially restricting scheduling options for Hepatitis B immunisation should it be introduced to the programme in the future. (2) Also, one alternative involves an earlier booster vaccination for Diphtheria, Tetanus, Polio, and Pertussis that includes a (later) booster for Hib, switching to a single vaccine booster for Men C and an earlier MMR booster.
Panovska-Griffiths, 2018	8/10	Cohort Study	UK	Transmission Modelling (for four diseases) and historic data synthesis (against the associated disease)/associating vaccination schedule to vaccine preventable disease.	(1) Describing and obtaining each set of VPDs a quantified relationship between the effective coverage against that disease and the residual burden of disease. (2) Defining the usage of these relationships to quantify the residual burden of disease associated with 4 distinct vaccine schedules relevant to the UK routine childhood vaccination programme.	(1) Four Immunisational schedules were explored differ by at most 19% in terms of the residual burden of disease expressed in QALYs loss. (2) The differences between the estimates of residual burden of disease associated with schedules A and B (9%) illustrate the potential benefits of scheduling vaccination to be completed at younger ages, with benefits driven by two effects within the model–younger vaccination being associated with slightly higher uptake and younger vaccination giving higher time-averaged protection among the study cohort.
*Study Characteristics of Vaccination Procurement and Distribution*						
**Study**	**Quality**	**Type of Study**	**Setting** **/(Country)**	**Population/(Subject)**	**Intervention/Method**	**Result/Outcomes**
Hardelid, 2016	8/10	Cohort study	The Health Improvement Network (THIN)/England and Wales	Preschool children aged 2- 4 years old	Encoding and analysing data that contains patients’ information entered into patient electronic records during patient consultation.	(1) 38.7% (95% CI 38.3% to 39.1%) of children were vaccinated against influenza. Children in the poorest deprivation quintile were 19% less likely to receive influenza vaccine than those in the wealthiest quintile (adjusted risk ratio (ARR) 0.81, 95%CI 0.77 to 0.86). (2) Children who received a timely first dose of measles-mumps-rubella vaccine were twice as likely to receive influenza vaccine (ARR 2.00 95% CI 1.87 to 2.13).
Petras, 2016	8/10	Cohort study	National Surveillance Programme/ Czech Republic	Children under 5 years old/analysis of immunisation coverage and IPD occurrence	Assessing the situation before and after routine immunisation over a two-year period, i.e., 2007–2008 (pre-immunisation period) and 2012– 2013 (post-immunisation period), respectively.	(1) In the child population there was an overall decline in IPD occurrence of 46.6% (95% CI 63.4–21.9) observed during the post- immunisation period. (2) There was even greater decrease of 71.6% (95% CI 50.4–83.8) in vaccinated children. The occurrence of 10 serotypes contained in both commercial vaccines also decreased in unvaccinated children by 61.4% (95% CI 14.5–82.6).
Grieco, 2020	8/10	Cross-sectional study	Modelling Framework/UK	Epidemiological Model of Influenza to estimate the beneficial mass immunisation	(1) An existing epidemiological model of influenza spread among the UK population to enable the evaluation of a large number of scenarios, each characterised by a unique combination of: the features of a mass immunization programme, the nature of the next influenza pandemic and the availability or otherwise of effective antiviral drugs with which to treat infected cases. (2) The output of the epidemiological model in a health economic analysis to estimate the net benefit of mass immunisation in that scenario. (3) Given the very large number of scenarios explored, we devised a compact visualisation of the model output to enable insights to be drawn about different preparedness policies. We describe these components of our work below	(1) One alternative combination of vaccines to the current set that did not violate our constraints that involves an earlier booster vaccination for Diphtheria, Tetanus, Polio, and Pertussis; includes a (later) booster for Hib, switching to a single vaccine booster for Men C and an earlier MMR booster 18% of children had received only DTaP-IPV-Hib-1 and -2 and no MMR-1 by age 15 months and were therefore included in the cohort under study. (2) By age 24 months among children in the included cohort, 26% had received both MMR-1 and DTaP-IPV-Hib-3, either simultaneously or at two separate visits; 12% of children had received only DTaP-IPV-Hib-3; 44% had received only MMR-1; and 18% received neither MMR-1 nor DTaP- IPV-Hib-3 before 24 months. In the <6 months sub-group, 95% received MMR-1 as recommended. children in the 6 months+ subgroup, only 7% received MMR-1 and DTaP- IPV-Hib-3 simultaneously.
*Study Characteristics of Combination/Multiple Vaccine Administration*						
**Study**	**Quality**	**Type of Study**	**Setting/(Country)**	**Population/** **(Subject)**	**Intervention/Method**	**Result/Outcomes**
Pedersen, 2020	8/10	Cohort study	Nationwide register-based study/Denmark	Children 15- missed MMR-1 and DTaP- IPV-Hib-3 doses	Assessing the compliance of the immunisation guidelines and the reasons for non-compliance with a focus on vaccination providers thru semi-structured telephone interviews with vaccination providers.	(1) The proportion of infants, children under 1 year old, receiving a hexavalent vaccine increased from 93.1% in 2017 to 98.6% in 2018, corresponding to an increase of VC against hepatitis B from around 92% in 2017 to 98% in 2018. VC for at least one dose of pneumococcal vaccine increased from 98.0% to 99.4%, and vaccine coverage for the first dose of meningococcal C vaccine increased from 39.3% to 75.7%. (2) This sharp increase in MenC VC translated into a dramatic decrease in the number of invasive MenC disease cases notified in infants through the mandatory notification system, from 17 cases on average during the 2012–16 period to four in 2018, all in non-vaccinated individuals. (3) This contrasts with the very limited decrease in incidence in individuals above 1 year of age in 2018.
*Study Characteristics of Improved Immunisation Timing and Intervals*						
**Study**	**Quality**	**Type of Study**	**Setting/(Country)**	**Population/(Subject)**	**Intervention/Method**	**Result/Outcomes**
Bielicki, 2012	8/10	Cohort study	Switzerland	Timing and timeliness of measles immunisations influence effective population vaccine coverage 0–3 years old	(1) Analysing time-to-event susceptibility to describe timing of measles immunisation. (2) Calculating effective vaccine coverage using an area under the curve approach.	(1) Taking into account the timing and timeliness of measles immunisation, children in our cohort spent on average 266 days (95% CI: 265.1–266.8) unvaccinated and susceptible to measles until their second birthday. (2) Of the susceptible days, 66.5% were spent susceptible due to the policy of recommending MCV1 for 12-month-olds and despite early uptake of MCV1 between 9 and 12 months by ∼20% in our cohort. (3) Conversely, 33.5% of susceptible days were due to delayed vaccinations.
Schweitzer, 2015	9/10	Cohort Study	Demographic and Health Surveys/Armenia and Kyrgyzstan	Children between 12 and 59 months of age for DTP vaccines and between 18 and 59 months assessing the up-to-date vaccination coverage	(1) Combining transmission modelling (for four diseases) and historic data synthesis (for eight diseases) to project, for each disease, the disease burden at different levels of effective coverage against the associated disease. (2) Determining the vector of effective coverage against each disease under three variations of the current childhood schedule using simulation model.	(1) The proportion of children in Armenia with correctly timed first DTP dose (DTP1) increased from 46% (2000) to 66% (2010). (2) In Kyrgystan, the proportion of correctly timed DTP1 increased from 75% (1997) to 87% (2012).
*Study Characteristics of Parental Education and Reminder*						
**Study**	**Quality**	**Type of Study**	**Setting** **/(Country)**	**Population/** **(Subject)**	**Intervention/Method**	**Result/Outcomes**
Napolitano, 2018	9/10	Cross-sectional study	Italy	Children aged 2 to 6 years	Questionnaires and Parent Attitudes about Childhood Vaccines Survey (PACV)	(1) Most important in determining parents’ vaccine hesitancy was to be not sure (OR D 16.14; 95% CI D 3.21–81.03) and uncertain in the pediatrician (OR D 3.56; 95% CI D 1.36–9.36). (2) Parents who were not sure (OR D 2.34; 95% CI D 1.27–4.31) and uncertain (OR D 2.09; 95% CI D 1.13–3.85) that to follow the recommended shot schedule is a good idea for their children, and those who were parents of first-born children (OR D 1.76; 95% CI D 1.12–2.76), compared to parents of second-born children, were more likely to need additional information about the childhood vaccinations.
Pavlopoulou, 2013	8/10	Cross-sectional study	Greece	10–65 months old	(1) A structured questionnaire completed by the investigators was used. Basic demographic data were collected from school registries on the day of school visit. (2) Detailed vaccination history and use of combination vaccines were obtained from vaccination booklets. (3) Parental/guardian attitudes towards immunisation and additional information were gathered on a second occasion by telephone interview	(1) Child’s age was strongly associated with incomplete vaccination with all vaccines (*p* < 0.001), while as immigrant status was a predictor of incomplete (*p* = 0.034) and delayed vaccination (*p* < 0.001) with traditional vaccines. (2) Increasing household size and higher maternal education were negatively associated with the receipt of all and newly licensed vaccines, respectively (*p* = 0.035).
*Study Characteristics of Surveillance Tool and Supplementary Immunisation Activity*						
**Study**	**Quality**	**Type of Study**	**Setting** **/(Country)**	**Population/** **(Subject)**	**Intervention/Method**	**Result/Outcomes**
Stoeckel, 2021	10/16	Ecological study	European Union	Relationship between vaccine hesitancy scores and uptake rates of DTP3, MCV1, and MCV2.	(1) Data on vaccine hesitancy comes from the Eurobarometer survey of Spring 2019. (2) Conducting face to face survey with probability samples from each EU member states except Luxembourg, Cyprus and Malta.	(1) We find vaccine hesitancy to be associated with DTP3 uptake (95% CI -3.658, -0.035), MCV1 uptake (95% CI -5.495, -0.779), and MCV2 uptake (95% CI -5.706, -0.264). (2) When taking uncertainty around regional estimates into account, we still find hesitancy to be related with DTP3 uptake (90% CI -3.139,-0.100), MCV1 uptake (95% CI -4.933, -0.186), and MCV2 uptake (95% CI -6.069, -0.520). (3) The results hold when hesitancy scores were calculated using MRP (DTP3: 95% CI -6.714, -0.074; MCV1: 95% CI -9.528, -1.169; MCV2: 95% CI -10.832, -0.282).
Edelstein, 2019	8/10	Cohort Study	United Kingdom of Great Britain and Northern Ireland	Children 24 months old and younger	Calculating the proportion of English population susceptible to measles using data from Primary Care and child health records.	(1) Measles susceptibility among people born between 1985 and 2016 was 4.6% (range: 1.2–9.2). (2) Of individuals who were eligible for the second MMR vaccine dose from October 1996 onwards, those born between 1998 and 2004 were in birth cohorts classified as not having sufficiently high level of immunity to prevent measles transmission.
*Study Characteristics of Information Technology*						
**Study**	**Quality**	**Type of Study**	**Setting** **/(Country)**	**Population/** **(Subject)**	**Intervention/Method**	**Result/Outcomes**
Alfonsi, 2012	8/10	Cross- sectional study	Level of computerization of immunization registers /Italy	Incorporating technology in childhood immunization program	(1) All regional coordinators for infectious diseases and vaccinations were contacted and asked to fill in a standarised online questionnaire. (2) It included 20 questions about thenumber of computerised LHU (Local Health Unit)s, use of different or the same software in the LHUs that were computerised, the name and basic characteristics of the software used. (3) Regional coordinators who reported having a single computerised regional register were asked by email or telephone about the characteristics of thesoftware used in the register, confidentiality issues, perspectives for future development and any aspects to be improved.	(1) 15 of the regions and 130 (83%) of LHUs are fully computerised, five regions are partially computerized and one does not use a computerised register. (2) Among the 15 fully computerised regions, eight use the same software in all LHUs, while the remaining seven use different software. In the five regions not fully computerised, the proportion of LHUs that are computerised ranges from 25% to 92% of the LHUs. (3) Eight of the 21 regions receive data every six months from the LHUs, four receive data in real time, three receive them quarterly and five yearly. (4) The capacity of the different systems to manage vaccination coverage data at regional level is very heterogeneous: of the 15 regions that are fully computerised, only six are able to calculate vaccine coverage automatically.
Rigby, 2020	High	Systematic Review and Meta- analyses	European Union	Electronic Health Record	(1) The Models of Child Health Appraised (MOCHA) project, aimed at assessing all aspects of primary healthcare for children in all EU and European Economic Area (EEA) countries. (2) Trillium II project, running from 2015 to 2019 [54] (https://trillium2.eu, 17 August 2022), promotes adoption of an International Patient Summary (IPS) as the means of transmitting unambiguous patient data across settings.	(1) The steps needed to promote immunisation holistic, child-centric, preventive child health services, in an efficient and sensitive manner using e-health support appropriately and innovatively. (2) The issues identified from the situation review need cross-sectoral and cross- stakeholder consideration so as to promote informed and effective approaches to strengthening child.

## Data Availability

If reasonably requested, data generated in this study can be obtained by contacting the first author, Ronan Lemwel Valdecantos.

## References

[1] Li X., Mukandavire C., Cucunubá Z.M., Echeverria Londono S., Abbas K., Clapham H.E., Jit M., Johnson H.L., Papadopoulos T., Vynnycky E. (2021). Estimating the health impact of vaccination against ten pathogens in 98 low-income and middle-income countries from 2000 to 2030: A modelling study. Lancet.

[2] Galles N.C., Liu P.Y., Updike R.L., Fullman N., Nguyen J., Rolfe S., Sbarra A.N., Schipp M.F., Marks A., Abady G.G. (2021). Measuring routine childhood vaccination coverage in 204 countries and territories, 1980–2019: A systematic analysis for the Global Burden of Disease Study 2020, Release 1. Lancet.

[3] World Health Organization (2019). The Global Vaccine Action Plan 2011–2020: Review and Lessons Learned: Strategic Advisory Group of Experts on Immunization.

[4] Muhoza P., Danovaro-Holliday M.C., Diallo M.S., Murphy P., Sodha S.V., Requejo J.H., Wallace A.S. (2021). Routine Vaccination Coverage—Worldwide, 2020. MMWR Morb. Mortal. Wkly. Rep..

[5] World Health Organization Immunization, Vaccines and Biologicals. https://apps.who.int/immunization_monitoring/globalsummary/timeseries/tscoveragemcv2.html.

[6] Vecchio A.L., Cambriglia M.D., Fedele M.C., Basile F.W., Chiatto F., del Giudice M.M., Guarino A. (2018). Determinants of low measles vaccination coverage in children living in an endemic area. Eur. J. Pediatr..

[7] Chiappini E., Parigi S., Galli L., Licari A., Brambilla I., Tosca M.A., Ciprandi G., Marseglia G. (2021). Impact that the COVID-19 pandemic on routine childhood vaccinations and challenges ahead: A narrative review. Acta Paediatr..

[8] DeSilva M.B., Haapala J., Vazquez-Benitez G., Daley M.F., Nordin J.D., Klein N.P., Henninger M.L., Williams J.T.B., Hambidge S.J., Jackson M.L. (2022). Association of the COVID-19 Pandemic with Routine Childhood Vaccination Rates and Proportion Up to Date with Vaccinations Across 8 US Health Systems in the Vaccine Safety Datalink. JAMA Pediatr..

[9] Esposito S., Principi N. (2008). Differences in vaccinations in European Union. Hum. Vaccines.

[10] Pelullo C.P., Marino S., Abuadili A.J.V., Signoriello G., Attena F. (2014). Is it reasonable to abandon obligatory vaccinations in Italy? A 2013 survey. Eurosurveillance.

[11] Loer K. (2019). Approaches and Instruments in Health Promotion and the Prevention of Diseases. Behavioural Policies for Health Promotion and Disease Prevention.

[12] Rigby M.J., Chronaki C.E., Deshpande S.S., Altorjai P., Brenner M., Blair M.E. (2020). European Union Initiatives in Child Immunization - The Need for Child Centricity, e-Health and Holistic Delivery. Eur J. Public Health.

[13] Wiese-Posselt M., Reiter S., Gilsdorf A., Krause G. (2009). Notwendigkeiten Und Hürden Einheitlicher Impfempfehlungen in Der Europäischen Union. Bundesgesundheitsblatt Gesundh. Gesundh..

[14] Blume S. (2006). Anti-Vaccination Movements and Their Interpretations. Soc. Sci. Med..

[15] Three Shots at Prevention: The HPV Vaccine and the Politics of Medicine’s. https://books.google.it/books?hl=en&lr=&id=_fBcYB4g5DkC&oi=fnd&pg=PA270&dq=L%C3%B6wy+I.+HPV+vaccination+in+context:+a+view+from+France.+In:+Wailoo+K,+Livingston+J,+Epstein+S,+Aronowitz+R,+editors.+The+HPV+vaccine+controversies.+New+Brunswick:+Rutgers+University+Press%3B+2010.+p.+270%E2%80%93&ots=ByWo6qLolE&sig=FUu153ZFxljqcYzyBREy_2C-gig&redir_esc=y#v=onepage&q&f=false.

[16] Bram J.T., Warwick-Clark B., Obeysekare E., Mehta K. (2015). Utilization and Monetization of Healthcare Data in Developing Countries. Big Data.

[17] Brown K.F., Kroll J.S., Hudson M.J., Ramsay M., Green J., Long S.J., Vincent C.A., Fraser G., Sevdalis N. (2010). Factors Underlying Parental Decisions about Combination Childhood Vaccinations Including MMR: A Systematic Review. Vaccine.

[18] Williams N., Woodward H., Majeed A., Saxena S. (2011). Primary care strategies to improve childhood immunisation uptake in developed countries: Systematic review. JRSM Short Rep..

[19] Henrikson N.B., Opel D.J., Grothaus L., Nelson J., Scrol A., Dunn J., Faubion T., Roberts M., Marcuse E.K., Grossman D.C. (2015). Physician Communication Training and Parental Vaccine Hesitancy: A Randomized Trial. Pediatrics.

[20] Turner N.M., Charania N.A., Chong A., Stewart J., Taylor L. (2017). The challenges and opportunities of translating best practice immunisation strategies among low performing general practices to reduce equity gaps in childhood immunisation coverage in New Zealand. BMC Nurs..

[21] Sadaf A., Richards J.L., Glanz J., Salmon D.A., Omer S.B. (2013). A systematic review of interventions for reducing parental vaccine refusal and vaccine hesitancy. Vaccine.

[22] Wilson S.L., Wiysonge C. (2020). Social media and vaccine hesitancy. BMJ Glob. Health.

[23] Nowak G.J., Gellin B.G., MacDonald N.E., Butler R. (2015). Addressing vaccine hesitancy: The potential value of commercial and social marketing principles and practices. Vaccine.

[24] Olson O., Berry C., Kumar N. (2020). Addressing Parental Vaccine Hesitancy towards Childhood Vaccines in the United States: A Systematic Literature Review of Communication Interventions and Strategies. Vaccines.

[25] Kumar D., Chandra R., Mathur M., Samdariya S., Kapoor N. (2016). Vaccine hesitancy: Understanding better to address better. Isr. J. Health Policy Res..

[26] Koshy E., Murray J., Bottle A., Sharland M., Saxena S. (2010). Impact of the seven-valent pneumococcal conjugate vaccination (PCV7) programme on childhood hospital admissions for bacterial pneumonia and empyema in England: National time-trends study, 1997–2008. Thorax.

[27] Derrough T., Olsson K., Gianfredi V., Simondon F., Heijbel H., Danielsson N., Kramarz P., Pastore-Celentano L. (2017). Immunisation Information Systems—Useful Tools for Monitoring Vaccination Programmes in EU/EEA Countries, 2016. Eurosurveillance.

[28] Fadda M., Depping M.K., Schulz P.J. (2015). Addressing Issues of Vaccination Literacy and Psychological Empowerment in the Measles-Mumps-Rubella (MMR) Vaccination Decision-Making: A Qualitative Study Infectious Disease Epidemiology. BMC Public Health.

[29] Liberati A., Altman D.G., Tetzlaff J., Mulrow C., Gøtzsche P.C., Ioannidis J.P.A., Clarke M., Devereaux P.J., Kleijnen J., Moher D. (2009). The PRISMA statement for reporting systematic reviews and meta-analyses of studies that evaluate health care interventions: Explanation and elaboration. J. Clin. Epidemiol..

[30] Rayyan Intelligent Systematic Review. https://www.rayyan.ai/.

[31] Wells G., Shea B., Robertson J., Peterson J., Welch V., Losos M., Tugwell P. (2011). The Newcastle-Ottawa Scale (NOS) for Assessing the Quality of Nonrandomized Studies in Meta-Analysis. Ott. Ott. Hosp. Res. Inst..

[32] Joanna Briggs Institute (2017). Checklist for Systematic Reviews and Research Syntheses Critical Appraisal Checklist for Systematic Reviews and Research Syntheses 2.

[33] AMSTAR—Assessing the Methodological Quality of Systematic Reviews. https://amstar.ca/Amstar_Checklist.php.

[34] Gofen A., Blomqvist P., Needham C.E., Warren K., Winblad U. (2017). Negotiated compliance at the street level: Personalizing immunization in England, Israel and Sweden. Public Adm..

[35] Lwembe S., Green S.A., Tanna N., Connor J., Valler C., Barnes R. (2016). A Qualitative Evaluation to Explore the Suitability, Fea-sibility and Acceptability of Using a “celebration Card” Intervention in Primary Care to Improve the Uptake of Childhood Vaccinations. BMC Fam. Pract..

[36] Gamlund E., Müller K.E., Paquet K.K., Solberg C.T. (2020). Mandatory childhood vaccination: Should Norway follow?. Etikk i Praksis Nord. J. Appl. Ethics.

[37] Bozzola E., Spina G., Russo R., Bozzola M., Corsello G., Villani A. (2018). Mandatory vaccinations in European countries, undocumented information, false news and the impact on vaccination uptake: The position of the Italian pediatric society. Ital. J. Pediatr..

[38] Lévy-Bruhl D., Desenclos J.C., Quelet S., Bourdillon F. (2018). Extension of French Vaccination Mandates: From the Recommendation of the Steering Committee of the Citizen Consultation on Vaccination to the Law. Eurosurveillance.

[39] Gianfredi V., D’Ancona F., Maraglino F., Cenci C., Iannazzo S. (2019). Polio and measles: Reasons of missed vaccination in Italy, 2015–2017. Ann. Ig. Med. Prev. Comunita.

[40] Martinot A., Leboucher B., Cohen R., Stahl J.-P., Subtil D., Pujol P., Lepetit H., Longfier L., Gaudelus J. (2021). Evolution between 2008 and 2018 of mothers’ perception regarding vaccination and infant vaccine coverage in France. Infect. Dis. Now.

[41] Panovska-Griffiths J., Crowe S., Pagel C., Shiri T., Grove P., Utley M. (2018). A method for evaluating and comparing immunisation schedules that cover multiple diseases: Illustrative application to the UK routine childhood vaccine schedule. Vaccine.

[42] Hardelid P., Rait G., Gilbert R., Petersen I. (2016). Factors associated with influenza vaccine uptake during a universal vaccination programme of preschool children in England and Wales: A cohort study. J. Epidemiol. Community Health.

[43] Petráš M., Adámková V. (2016). Epidemiology of Invasive Pneumococcal Disease in Czech Children under 5 Years of Age after Routine Immunisation. Cent. Eur. J. Public Health.

[44] Grieco L., Panovska-Griffiths J., van Leeuwen E., Grove P., Utley M. (2020). Exploring the role of mass immunisation in influenza pandemic preparedness: A modelling study for the UK context. Vaccine.

[45] Pedersen K.B., Holck M.E., Jensen A.K., Suppli C.H., Benn C.S., Krause T.G., Sørup S. (2018). How are children who are delayed in the Childhood Vaccination Programme vaccinated: A nationwide register-based cohort study of Danish children aged 15–24 months and semi-structured interviews with vaccination providers. Scand. J. Public Health.

[46] Bielicki J.A., Achermann R., Berger C. (2012). Timing of Measles Immunization and Effective Population Vaccine Coverage. Pediatrics.

[47] Schweitzer A., Krause G., Pessler F., Akmatov M.K. (2015). Improved coverage and timing of childhood vaccinations in two post-Soviet countries, Armenia and Kyrgyzstan. BMC Public Health.

[48] Napolitano F., D’Alessandro A., Angelillo I.F. (2018). Investigating Italian parents’ vaccine hesitancy: A cross-sectional survey. Hum. Vaccines Immunother..

[49] Pavlopoulou I.D., Michail K.A., Samoli E., Tsiftis G., Tsoumakas K. (2013). Immunization coverage and predictive factors for complete and age-appropriate vaccination among preschoolers in Athens, Greece: A cross-sectional study. BMC Public Health.

[50] Stoeckel F., Carter C., Lyons B.A., Reifler J. (2021). Association of vaccine hesitancy and immunization coverage rates in the European Union. Vaccine.

[51] Edelstein M., White J., Bukasa A., Saliba V., Ramsay M.E. (2019). Triangulation of measles vaccination data in the United Kingdom of Great Britain and Northern Ireland. Bull. World Health Organ..

[52] Alfonsi V., D’Ancona F., Rota M.C., Giambi C., Ranghiasci A., Iannazzo S. (2012). Regional Coordinators for Infectious Diseases and Collective Regional coordinators for infectious diseases and vaccinations Immunisation registers in Italy: A patchwork of computerisation. Eurosurveillance.

[53] Crowe S., Utley M., Walker G., Panovska-Griffiths J., Grove P., Pagel C. (2015). A novel approach to evaluating the UK childhood immunisation schedule: Estimating the effective coverage vector across the entire vaccine programme. BMC Infect. Dis..

[54] Trillium II. https://trillium2.eu/.

[55] Glanz J.M., Kraus C.R., Daley M.F. (2015). Addressing Parental Vaccine Concerns: Engagement, Balance, and Timing. PLoS Biol..

[56] Trentini F., Poletti P., Melegaro A., Merler S. (2019). The introduction of ‘No jab, No school’ policy and the refinement of measles immunisation strategies in high-income countries. BMC Med..

[57] MacDonald N.E., Harmon S., Dube E., Steenbeek A., Crowcroft N., Opel D.J., Faour D., Leask J., Butler R. (2018). Mandatory infant & childhood immunization: Rationales, issues and knowledge gaps. Vaccine.

[58] Charting Mandatory Childhood Vaccination Policies Worldwide|Elsevier Enhanced Reader. https://reader.elsevier.com/reader/sd/pii/S0264410X21005478?token=0FB3D24C6C236210BCD2D8FEFA34AD340958D56343033E3FECB1AA06EA91056F374A722CE116A4F97484704307341EA7&originRegion=eu-west-1&originCreation=20220110091743.

[59] Waight P.A., Andrews N.J., Ladhani S.N., Sheppard C.L., Slack M.P.E., Miller E. (2015). Effect of the 13-valent pneumococcal conjugate vaccine on invasive pneumococcal disease in England and Wales 4 years after its introduction: An observational cohort study. Lancet Infect. Dis..

[60] Saeterdal I., Lewin S., Austvoll-Dahlgren A., Glenton C., Munabi-Babigumira S. (2014). Interventions aimed at communities to inform and/or educate about early childhood vaccination. Cochrane Database Syst. Rev..

[61] Stockwell M.S., Fiks A.G. (2013). Utilizing health information technology to improve vaccine communication and coverage. Hum. Vaccines Immunother..

[62] Chantler T., Karafillakis E., Wodajo S., Demissie S.D., Sile B., Mohammed S., Olorunsaiye C., Landegger J., Larson H.J. (2018). ‘We All Work Together to Vaccinate the Child’: A Formative Evaluation of a Community-Engagement Strategy Aimed at Closing the Immunization Gap in North-West Ethiopia. Int. J. Environ. Res. Public Health.

[63] Vaz O.M., Ellingson M.K., Weiss P., Jenness S.M., Bardají A., Bednarczyk R.A., Omer S.B. (2020). Mandatory Vaccination in Europe. Pediatrics.

[64] Miron V.D., Toma A.R., Filimon C., Bar G., Craiu M. (2022). Optional Vaccines in Children—Knowledge, Attitudes, and Practices in Romanian Parents. Vaccines.

[65] O’Doherty K.C., Crann S., Bucci L.M., Burgess M.M., Chauhan A., Goldenberg M.J., McMurtry C.M., White J., Willison D.J. (2021). Deliberation on Childhood Vaccination in Canada: Public Input on Ethical Trade-Offs in Vaccination Policy. AJOB Empir. Bioeth..

[66] Webster P.C. (2011). Go local, European review of electronic health records advises. Can. Med Assoc. J..

[67] Global Diffusion of EHealth: Making Universal Health Coverage Achievable—World Health Organization. https://books.google.it/books?hl=en&lr=&id=MnOyDwAAQBAJ&oi=fnd&pg=PP4&dq=The+ability+of+various+systems+to+manage+vaccination+coverage+data+at+the+regional+level+varies+greatly%3B+only+six+of+the+15+completely+computerized+regions+can+calculate+vaccine+coverage+automatically&ots=agT3EWjDvj&sig=QBaIRxv6xeDwwIzLNZTPPqdgyGQ&redir_esc=y#v=onepage&q&f=false.

[68] Marshall S., Sahm L.J., Moore A.C. (2016). Microneedle technology for immunisation: Perception, acceptability and suitability for paediatric use. Vaccine.

[69] Harvey H., Reissland N., Mason J. (2015). Parental reminder, recall and educational interventions to improve early childhood immunisation uptake: A systematic review and meta-analysis. Vaccine.

[70] Lombardi N., Crescioli G., Bettiol A., Tuccori M., Rossi M., Bonaiuti R., Ravaldi C., Levi M., Mugelli A., Ricci S. (2019). Vaccines Safety in Children and in General Population: A Pharmacovigilance Study on Adverse Events Following Anti-Infective Vaccination in Italy. Front. Pharmacol..

[71] Hu Y., Chen Y., Wang Y., Liang H. (2017). Evaluation of potentially achievable vaccination coverage of the second dose of measles containing vaccine with simultaneous administration and risk factors for missed opportunities among children in Zhejiang province, east China. Hum. Vaccines Immunother..

[72] Syiroj A.T.R., Pardosi J.F., Heywood A.E. (2019). Exploring parents’ reasons for incomplete childhood immunisation in Indonesia. Vaccine.

[73] Syed Y.Y. (2016). DTaP5-HB-IPV-Hib Vaccine (Vaxelis®): A Review of its Use in Primary and Booster Vaccination. Pediatr. Drugs.

[74] Bulula N., Mwiru D.P., Swalehe O., Mori A.T. (2020). Vaccine storage and distribution between expanded program on immunization and medical store department in Tanzania: A cost-minimization analysis. Vaccine.

[75] Wang W., Wang Y., Wang Y., Yan F., Wang N., Fu C. (2021). Vaccine bidding, procurement and distribution management practices in mainland China: A nationwide study. Vaccine.

[76] Decouttere C., Vandaele N., De Boeck K., Banzimana S. (2021). A Systems-Based Framework for Immunisation System Design: Six Loops, Three Flows, Two Paradigms. Health Syst..

[77] Danovaro-Holliday M.C., Dansereau E., Rhoda D.A., Brown D.W., Cutts F.T., Gacic-Dobo M. (2018). Collecting and Using Reliable Vaccination Coverage Survey Estimates: Summary and Recommendations from the “Meeting to Share Lessons Learnt from the Roll-out of the Updated WHO Vaccination Coverage Cluster Survey Reference Manual and to Set an Operational Research Agenda around Vaccination Coverage Surveys” Geneva, 18–21 April 2017. Vaccine.

[78] World Health Organization (2019). 2019 Intervention Guidebook Intervention Guidebook for Implementing and Monitoring Activities to Reduce Missed Opportunities for Vaccination.

[79] Cutts F.T., Claquin P., Danovaro-Holliday M.C., Rhoda D.A. (2016). Monitoring vaccination coverage: Defining the role of surveys. Vaccine.

[80] Opel D.J., Marcuse E.K. (2013). Rethinking vaccine policy making in an era of vaccine hesitancy: Time to rebuild, not remodel?. Hum. Vaccines Immunother..

